# Single-Step Synthesis of Ag Hexagonal Nanoplate-Decorated Reduced Graphene Oxide and Its Cytotoxicity Studies

**DOI:** 10.1155/2023/4466394

**Published:** 2023-07-01

**Authors:** Gnanaprakasam Periyasamy, Selvaraju Thangavelu, Saravanan Muthupandian

**Affiliations:** ^1^Department of Chemistry, Saveetha School of Engineering, Saveetha Institute of Medical and Technical Sciences (SIMATS), Chennai 60007, India; ^2^Department of Chemistry, Bharathiar University, Coimbatore 641 046, India; ^3^Department of Pharmacology, AMR and Nanomedicine Laboratory, Center for Transdisciplinary Research, Saveetha Dental College, Saveetha Institute of Medical and Technical Sciences (SIMATS), Chennai 60007, India

## Abstract

Graphene-based Ag nanocomposites are of specific interest because of their unique properties and applications, especially in the field of cytotoxicity. However, developing a simple method to synthesize reduced graphene oxide (rGO)/silver hexagonal nanoplate (Ag HNPT) (rGO–Ag HNPT) nanocomposites with well-defined morphology has been believed to be a major challenge. In this work, a facile, robust, and single-step synthesis method was developed to prepare silver-graphene (rGO–Ag HNPT) nanocomposites with hexagonal-structured silver nanoplates without any templates. The primary characterizations of the synthesized nanocomposite were done using a UV-visible spectrophotometer, X-ray diffraction (XRD), and Raman spectroscopy. The formation of uniformed hexagonal-shaped Ag nanoplates was confirmed by high-resolution transmission electron microscopy (HR-TEM), and the elemental composition was confirmed using energy dispersive X-ray analysis (EDX). With SiHa cervical cancer cells, the short-term *in vitro* cytotoxicity of the as-synthesized rGO–Ag HNPTs was evaluated by the 3-(4,5-dimethylthiazol-2-yl)-2,5-diphenyltetrazolium bromide (MTT) assay. The anticancer response of the rGO–Ag HNPTs was investigated using an MTT assay.

## 1. Introduction

Over the last several years, silver nanoparticles (AgNPs) have attracted substantial interest among researchers due to their catalytic [1, 2], antimicrobial [3, 4], and antibacterial properties [5] as well as their optical [6], plasmonic [6], thermal [7], and electronic properties [8]. AgNPs with different shapes have unique electrical and optical properties when compared to the usual spherical sizes [9, 10]. Due to their shape-dependent properties, novel morphologies like nanorods (NRDs), triangles, and nanoplates (NPTs) can be applied as an improved biosensor and optoelectronic material [11, 12]. Jin et al. developed a photoinduced chemical reaction method to produce Ag nanoplates for the first time [13]. Other than photoirradiation methods [14], various other approaches such as the thermal process [15], microwave [16], and microemulsion methods [17] have also been reported, but the chemical reduction method is believed to be an efficient and ideal method for the synthesis of NRDs, NPTs, etc. [18]. In general, the kinetically controlled synthesis of NRDs, triangles, and hexagonal nanoplates (HNPTs) using chemical reduction methods will occur in two steps: (i) the formation of small seed particles and (ii) uniform growth of the aforementioned nanostructure with the help of suitable template molecules. Morphologies of the nanostructure could be directed by changing the concentration of precursors and template molecules [19, 20]. Several template molecules or stabilizing agents such as poly (N-vinyl-2-pyrrolidone) (PVP) [15], cetyltrimethylammonium bromide (CTAB) [21], and a mixture of PVP and polyethylene glycol (PEG) [22] have been reported for the synthesis of NPTs. PVP has proven to be the best stabilizing agent in the chemical reduction approach that guides the growth of Ag NPTs. Limited works in the literature are available on the single-step process for the synthesis of the triangle or hexagonal NPTs. For example, Xiong et al. synthesized triangular-shaped Ag NPTs with PVP as the reductant and stabilizer in addition to Ag. They also reported the formation of Pd, Au, and Pt NPTs with triangular and hexagonal shapes. [23]. Lim et al. demonstrated the synthesis of Ag hexagonal nanoplates (Ag-HNPTs) using PVP with a thermal treatment method [24]. Yu et al. developed a two-step protocol for synthesizing triangular Ag NPTs at low temperatures [25]. To the best of our knowledge, there are no reports available for the single-step synthesis of graphene-based Ag-HNPT composites. Reduced graphene oxide- (rGO-) based nanosheets tend to prevent the aggregation of metal nanoparticles, and the nanosheets of the negatively charged rGO could serve as a stable base to allow metal ions to attach and grow into the nanostructures of choice. The existence of an oxygen functional group on rGO nanosheet surfaces stimulates the growth and stabilization of the metal nanostructures [26, 27]. The Ag-HNPTs decorated on the surface of rGO nanosheets can retain their catalytic and biocatalytic activities even after the exploration of the catalytic reaction because of the stable support given by the graphene backbone [27]. All these factors inspired us to synthesize the Ag hexagonal NPTs (Ag-HNPTs) that were incorporated on rGO in a single step without the need for any external templates. Recent research has focused on graphene-based metal nanoparticles designed for biomedical applications [28] such as tissue engineering, gene transport, drug delivery systems, bioimaging, cancer therapy, and biosensors. Sawan et al. synthesized graphene functionalized AgNPs and fabricated orthodontic brackets with high mechanical strength and antimicrobial properties. [29]. Au- and Ag-decorated rGO was fabricated by Gnanasekar et al., and the cytotoxicity effect of the composite against two breast carcinoma cell lines (MDA-MD-231 and MDA-MD-468) was evaluated and identified, as the synthesized material has less toxicity and could be utilized for breast cancer therapy [30]. Ghosh et al. evaluated the anticancer and antimicrobial properties of the GO supramolecular hydrogel network with AgNPs and discovered that the synthesized material has excellent anticancer activity against human breast cancer cells and is recognized as a great replacement for intracellular image analysis due to its luminescent nature [31]. Palai et al. prepared amino-PEGylated Ag NP-decorated graphene support in an environmentally friendly manner, and the synthesized substance precisely targets cancer cells with specific cytotoxic effects [32]. As a result, the author asserted that the produced composite could be a good nanocargo for controlled and targeted drug delivery in cancer therapy.

Cancer is a common term disease associated with the growth of abnormal cells uncontrollably and tends to spread to other parts of the body and damage normal cells [33]. Cancer can begin practically any place in the human body; the most common form of cancer in women is cervical cancer, where abnormal growth of cells in the cervix occurs [34]. According to data, 5,28,000 new cases of cervical cancer were reported in 2012, with 2,66,000 deaths. The protein of the human papillomavirus (HPV E7) is the viral oncoprotein required for neoplastic progression in HPV infection-related cancers [35]. For the successful immortalization of human keratinocytes, the collaborative impact of HR E6 and E7 proteins is sufficient, and for the preservation of HPV cancer cell lines, the two forms of oncoproteins are important [36]. Cancer is, however, a secondary consequence of the E6 and E7 functions that are important to the viral life cycle. For vegetative amplification of the viral genome in the differentiated stratified epithelium, the function of the E7 protein is important [37]. The E7 protein is expressed in a differentiation-dependent manner from the viral upstream regulatory region (URR) and induces suprabasal keratinocyte S-phase reentry. In the basal stratum of the epidermis, cell-cycle-dependent DNA replication does not produce a large copy number of HPV plasmids. Upon differentiation, E7 restores the S-phase state to permanently withdraw from the cell cycle in terminally differentiating keratinocytes. By S-phase reentry, the E7 protein produces a replicative environment in which the viral genome uses the host DNA replication machinery for vegetative multiplication [38, 39].

HPV E7 is an acidic protein with a molecular weight of 11 kDa and 100 amino acids. Many non-HPV viral oncoproteins have similar sequences to HPV E7 which include Epstein-Barr virus nuclear antigen 3C, human cytomegalovirus pp71, SV40 large T antigen, Merkel cell polyomavirus large T antigen, and adenovirus E1A [39]. Cervical cancer occurs most often in the Indian subcontinent due to SiHa cell lines [40]. There are a variety of treatments for cancer cells, including chemotherapy, surgery, and radiotherapy [41]. There has been some evidence that these methods have adverse side effects, including abdominal discomfort, hair loss, constipation, fatigue, diarrhea, and infertility [42]. Nanomaterials with graphene-based therapy and diagnosis have improved the efficacy of cancer treatment and addressed the limitations of traditional protocols. A one-step process without the involvement of any template molecules was demonstrated in this study to synthesize the rGO nanosheets decorated with Ag hexagonal nanoplates, and their anticancer activity on SiHa cervical cancer cells was determined using the 3-(4,5-dimethylthiazol-2-yl)-2,5-diphenyltetrazolium bromide (MTT) assay.

## 2. Experimental Section

### 2.1. Materials

Graphite flakes (300 mesh) were acquired from Alfa Aesar, U.K., sodium nitrate (NaNO_3_), potassium permanganate (KMnO_4_), sulphuric acid (H_2_SO_4_), polyvinyl pyrrolidone (PVP), and hydrochloric acid (HCl) were procured from Aldrich, U.S.A., silver nitrate (AgNO_3_), hydrazine hydrochloride (N_2_H_6_Cl_2_), potassium hydroxide (KOH), hydrogen peroxide (H_2_O_2_), and aqueous NH_3_ were obtained from Merck, India. All of the solutions were made with double-distilled water.

### 2.2. Synthesis of Ag Hexagonal NPT-Decorated rGO (rGO–Ag HNPT)

The starting material for the manufacture of rGO–Ag HNPTs is graphene oxide dispersion in water. The detailed procedure for graphene oxide production has been published [43]. Through sonication, a stable GO dispersion (40 mg/40 ml) was obtained; 320 mg of PVP was mixed with the GO dispersion (GO/PVP) and stirred for 12 hours to enhance the stability of the dispersion by preventing the aggregation of each graphene sheet. For a typical rGO–Ag HNPT synthesis, the precalculated amount of GO dispersion (GO/PVP) (40 ml) was mixed with AgNO_3_ solution (20 ml, 1 mm) and agitated for 30 minutes. The synthesis reaction was completed by chemical reduction of the above GO dispersion by adding aqueous NH_3_ (4.8 ml) and hydrazine hydrochloride (600 *μ*l) in the dispersion at 80°C for 3 h and 30 min. The mixture's color shifted from brown to blackish grey, indicating that rGO–Ag HNPTs were formed. To eliminate surplus reducing agents, the resulting mixture was centrifuged and rinsed multiple times with water. Finally, the centrifuged material was indicated as rGO–Ag HNPTs after drying in a hot air oven at 50°C.

### 2.3. Characterization

The absorption spectra of rGO–Ag HNPTs in the UV-visible range were measured utilizing Shimadzu UV 2600, Japan. The Cu-K radiation (=1.5418) was used to record the XRD diffraction patterns on a Shimadzu XRD 6000 (Japan). HRTEM (JEOL JEM-2100, UK) studies were used to investigate the surface morphology of the as-synthesized material. EDAX analysis proved the presence of Ag on the surface of graphene sheets (Bruker Nano GmbH, Germany). Using a HORIBA Jobin, LabRAM HR Raman spectroscopy integrated with argon ion (514 nm) laser excitation source, the investigation of the order nature of graphene nanosheets (rGO) was performed.

## 3. Results and Discussion

### 3.1. Synthesis of rGO–Ag HNPTs

**Scheme 1:**
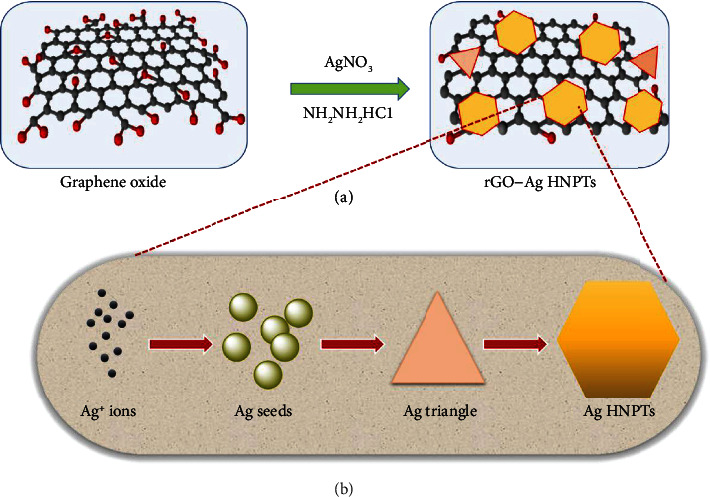
The synthesis of rGO–Ag HNPTs (a) and the mechanism of Ag HNPT generation on the surface of rGO are depicted schematically (b).

A schematic representation of the one-pot, template-free synthesis of GO into rGO–Ag HNPTs is shown in Scheme 1(a). The detailed mechanism of the formation of Ag HNPTs from Ag precursors on the rGO surface is illustrated in Scheme 1(b).

### 3.2. UV-Visible Characterization

**Figure 1:**
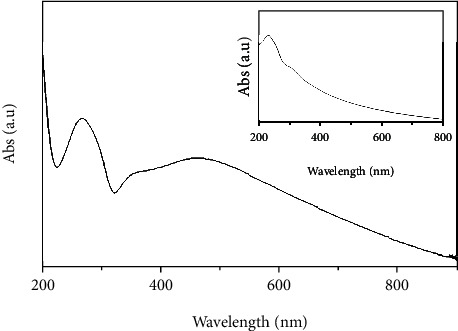
UV-visible absorption spectrum of rGO–Ag HNPTs, with a GO absorption spectrum inset.

The UV-visible spectrophotometer was utilized to investigate the effective conversion of GO into rGO–Ag HNPTs.  Figure 1 (inset) displays the absorbance spectrum of GO, which has a peak at 230 nm and a slight hump at 303 nm, which correspond to the aromatic C = C and carbonyl groups as the result of *π*⟶*π*^∗^ and n⟶*π*^∗^ transitions.  Figure 1 depicts the UV spectroscopy experiments for rGO–Ag HNPTs, which shows a strong peak at 264 nm, indicating that oxygen functional groups in GO were successfully reduced to rGO, forming a graphitic *π* conjugation network. The absorption at 460 nm was also noticed due to the surface plasmon resonance (SPR) of Ag NPTs. The UV spectra of 2D nanomaterials such as NPTs and NRDs often have two SPR peaks, because electrons oscillate transversely and longitudinally. However, in the current investigation, the rGO–Ag HNPTs exhibit a single broad SPR peak at 460 nm as a consolidation of both oscillations.

### 3.3. HRTEM and EDX Analyses

**Figure 2:**
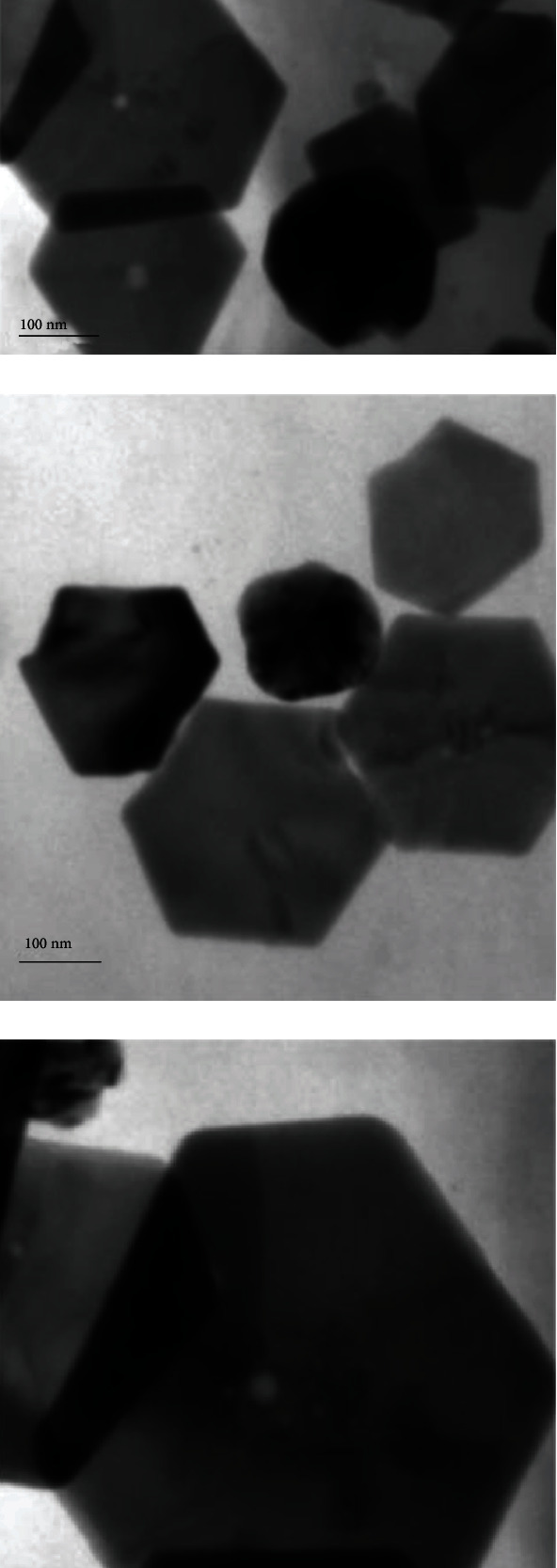
HR−TEM images show the presence of uniform Ag hexagonal nanoplates on the rGO surface with various magnifications.

**Figure 3:**
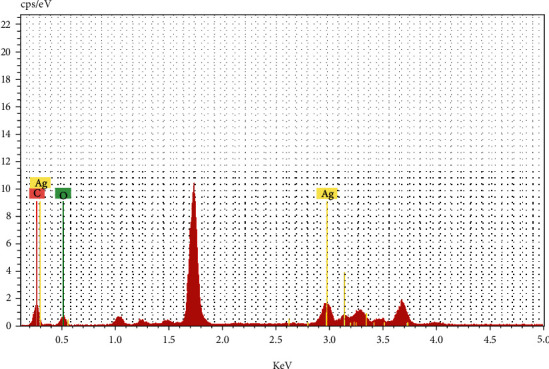
EDX diagram for analyzing the elemental presence of Ag and carbon in rGO–Ag HNPT nanocomposite.

HRTEM analysis was used to investigate the surface morphology of the NPTs.  Figure 2 depicts the decorating of homogeneous Ag HNPTs on rGO nanosheets. In addition to the distinct HNPTs, intermediates like spherical and triangle NPs were also discovered (Figures 2(c)–2(e)). This reveals the development of HNPTs on the rGO surface taking place via Ag seeds and subsequently by Ag nanotriangles. Reaction kinetics of the conversion of Ag seed into the nanoplates should be slow enough for the uniform and successful growth of the nanoplates (triangles and hexagonal plates). Herein, PVP acts as a colloidal stabilizer and also adjusts the rate of reduction of Ag+ions, resulting in the effective formation of Ag HNPTs. The crystal lattice fringes are clearly visible in the magnified HRTEM images of the Ag HNPTs (Figures 2(h) and 2(i)), and the EDX spectra confirmed the presence of Ag in the synthesized rGO–Ag HNPTs (Figure 3).

### 3.4. XRD Analysis

**Figure 4:**
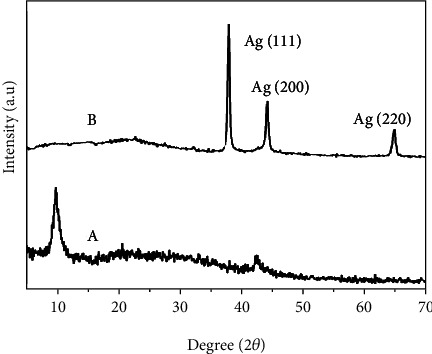
Typical XRD patterns of GO (a) and rGO–Ag HNPTs (b).

The crystal structure of the GO and rGO–Ag HNPTs was investigated using XRD patterns (Figure 4). The strong peak at 10.14° appears for GO (Figure 4(a)) and corresponds to the exfoliated graphene sheets with an interlayer distance of 8.3 nm. In a comparison among two XRD patterns, the sharp peak at 10.14° corresponds to the GO in Figure 4(a) is missing in Figure 4(b) (rGO–Ag HNPTs) indicating the complete reduction of the functional groups of GO. The sharp peaks of Ag appeared at 37.94°, 44.13°, and 64.35° assigned to the lattice planes of (111), (200), and (220) face-centered cubic (fcc) Ag HNPTs (JCPDS 89-3722). The comparison among the intensities of (111), (200), and (220), plane (111) is very intensified than others which inferred that the synthesized rGO–Ag HNPTs are majorly made up of (111) planes. Aside from the strong peaks, a tiny peak at 23.71° was also obtained, which is responsible for the graphitic structure. The complete reduction of GO into rGO is confirmed as a result of the development of the new peak and the removal of the GO peak (10.14°). Aside from the sharp peaks, a minor curvature at 23.71° was observed, which is primarily responsible for the graphitic structure. As a result, the disappearance of the GO peak (10.14°) and the formation of a new peak at 23.71° confirm the complete conversion of GO to rGO.

### 3.5. Raman Analysis

**Figure 5:**
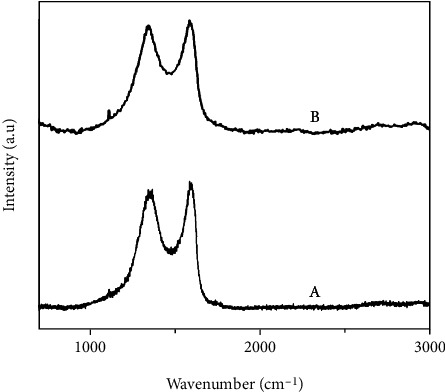
The Raman spectra of GO (a) and rGO–Ag HNPTs (b).

To study the orderliness and the hybridization of carbon in GO and rGO–Ag HNPTs, Raman spectra were carried out and the respective sp^3^ and sp^2^ domains of carbon are obtained at the peak of 1348 cm^−1^ (*D*-band) and 1588 cm^−1^ (*G*-band) (Figures 5(a) and 5(b) a, b). The intensity of the ratio of ID/IG reveals the orderliness of the graphene nanosheets. The ID/IG ratios for GO and rGO–Ag HNPTs were 0.94 and 0.95, respectively. The synthesized graphene sheets have fewer defects and a more stable sp^2^ domain. Furthermore, the sp^2^ domain stays stable (fewer defects) even when GO is reduced to rGO–Ag HNPTs. As a result, the orderly nature of graphene nanosheets and good electrical conductivity is maintained after the development of Ag HNPTs.

Variety of parameters, such as precursor concentration, reducing agent, capping agent, temperature, and reaction time, all influence the form of the NPs [44]. The standard procedure for preparing NRDs, triangles, and NPTs is a two-step process. Seed preparation is primarily done using mild reducing agents such as ascorbic acid and sodium citrate. Second, using the proper template or directing agents; the seed is nucleated into the relevant one- or two-dimensional NPs [44, 45]. PVP is well known as an effective colloidal stabilizer and directing agent in the formation of NPTs [46]. This paper elucidates the detailed mechanism of rGO–Ag HNPT synthesis as follows.

Despite the fact that hydrazine (*E*° = −0.230 V) is a stronger reducing agent than many well-known reducers such as ascorbic acid and sodium citrate, it was used as a reducing agent to simultaneously reduce the GO and Ag precursor in this study. The oxygen functional groups of GO consumed a major amount of hydrazine molecules and reduced into the rGO; hence, the availability of the hydrazine molecule to reduce Ag will be meager; hence, the strong hydrazine will behave like the weak reducing agent similar to ascorbic acid or sodium citrate and helped to form Ag seed on the rGO surface. To avoid the agglomeration of the graphene nanosheets due to the *π*–*π* interaction, PVP was introduced into GO which acted as the colloid stabilizer. Fortunately, the PVP on the GO surface promotes the formation of Ag HNPTs by stimulating the nucleation of Ag seeds on the rGO, resulting in the formation of hexagonal nanoplates assigned to the high-intensity lattice planes of (111) as face-centered cubic (fcc), as confirmed by the XRD pattern. As the structures of NPTs were recognized, the PVP played a dual role as a colloidal stabilizer and also the directing agent to induce the growth of Ag seeds. The cytotoxic activity of the rGO–Ag HNPTs against the SiHa cell line (cervical cancer) was also evaluated using the MTT assay.

### 3.6. MTT Assay for Cytotoxicity Investigations

**Figure 6:**
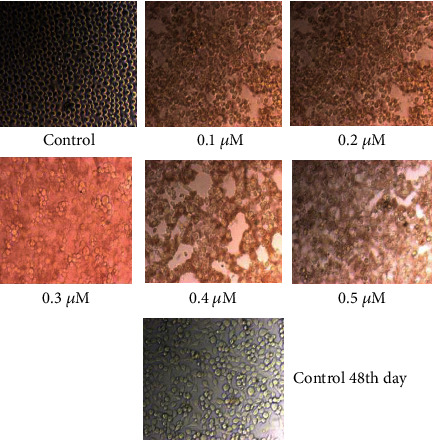
Morphological changes of SiHa cells with control, and the different concentration of rGO–Ag HNPTs (0.1 *μ*m to 0.5 *μ*m).

**Figure 7:**
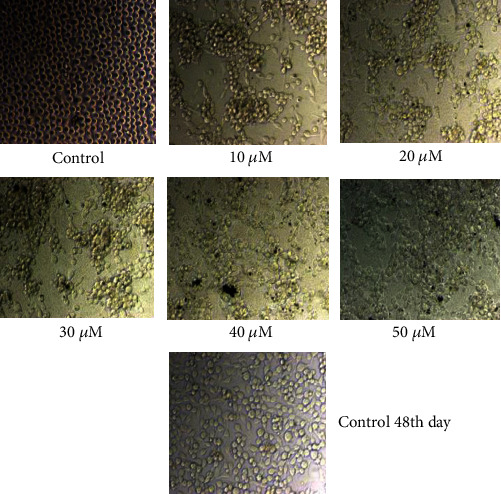
Morphological changes of SiHa cells with control, and the different concentration of rGO–Ag HNPTs (10 *μ*m to 50 *μ*m).

**Figure 8:**
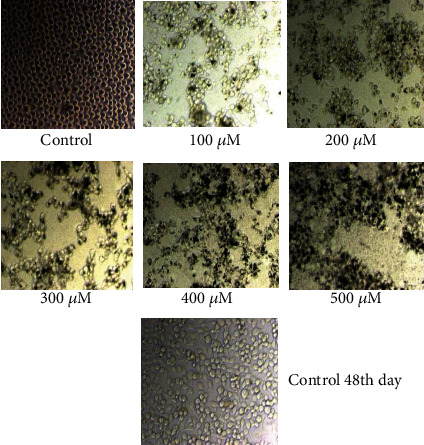
Morphological changes of SiHa cells with control, and different concentration of rGO–Ag HNPTs (100 *μ*m to 500 *μ*m).

The MTT assay is a straightforward colorimetric experiment used to determine cell viability, cytotoxicity, or proliferation. In this experiment, live cells are evaluated to convert 3-4,5-dimethylthiazol-2-yl-2,5-diphenyl tetrazolium bromide (MTT) to purple insoluble formazan. The formed formazan crystals are dissolved in an organic solvent such as dimethyl sulfoxide (DMSO), and the optical density of the solution is measured using a multiwall spectrophotometer. In this study, the MTT assay was used to assess the cytotoxicity of rGO–Ag HNPTs in cervical cancer cells. The rGO–Ag HNPTs were screened based on their score for testing their anticancer effect on the SiHa cell line using the MTT assay. Yellow tetrazolium MTT was reduced by metabolically active cells, in part by dehydrogenase enzymes, to form reducing equivalents such as NADH and NADPH. The resulting intracellular purple formazan was solubilized and measured using spectrophotometric methods. As a result, the concept was used to test the cytotoxic effect of rGO–Ag HNPTs as medicine. If the medicine is pharmacologically active, it should actively block cancer cell proliferation, and the resulting activity should increase linearly with concentration. As a result, the treated cells generate less formazan crystal than the untreated ones. To evaluate *in vitro* cytotoxicity, confluent cells were treated with various concentrations of rGO–Ag HNPTs as the medication (0.1-0.5 M, 10-50 M, 100-500 M) in DMSO for 48 hours. DMSO is a polar aprotic solvent that dissolves both polar and nonpolar molecules, as well as a well-known antioxidant that is widely employed in biomedical research. Furthermore, DMSO control trials were maintained to demonstrate the feasibility of interfering with rGO–Ag HNPTs. The cells were monitored for morphological changes every 24 hours. In cancer cells, cell necrosis, the primary event of cytotoxicity, occurs when cells enlarge rapidly, lose membrane integrity, and shut down metabolism. As a result, the cancer cells disappear quickly by initiating programmed cell death known as apoptosis, based on the results obtained from the microscopically seen figures (Figures 6–8). Morphological investigation indicated that the produced Ag HNPTs on the rGO surface have an excellent cytotoxic impact for usage as a possible anticancer medication. It was discovered that the lowest concentration of rGO–Ag HNPTs has the significant cytotoxicity activity. The higher concentrated rGO–Ag HNPTs are riskier than the lower concentrated nanocomposites to the aggregation between the graphene nanosheets, and the MTT assay clearly showed the molecular performance.

**Figure 9:**
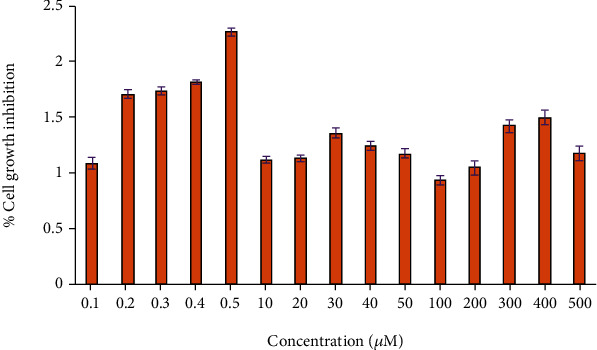
The MTT test was used to measure the viability of SiHa cells at dosages of rGO–Ag HNPTs ranging from 0.1 to 500 *μ*m.

**Figure 10:**
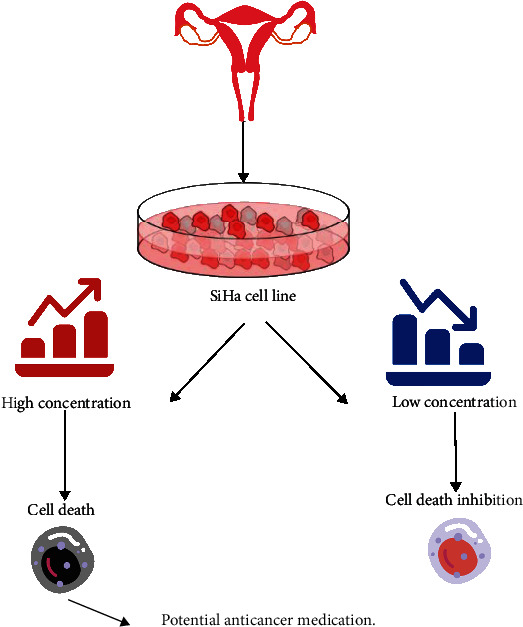
Mechanism of controlling the growth of inhibition of SiHa cell treated with rGO–Ag HNPTs.

The calculation of half maximal inhibitory concentration would be used to measure the effectiveness of the anticancer reaction in inhibiting the specific biological function (IC_50_). The IC_50_ value of the rGO–Ag HNPTs was calculated using the dose-response curve (Figure 9). A plot was made between the percentage of cell growth inhibition and the concentration of rGO–Ag HNPTs based on the analysis of the data to determine the morphological changes in the cell with regard to the concentration of the nanocomposites. In contrast to other concentrated nanocomposites, Figure 9 shows the lowest concentrated nanocomposites with the highest activity and lowest IC_50_ value. Last but not least, a research of IC_50_ values showed that the best concentration of rGO–Ag HNPTs for preventing cell growth is 0.5 M. The IC_50_ values for high concentrations, such as 100 M rGO–Ag HNPTs, were incredibly low. Overall, the research demonstrates that the HPV16 E7 protein is a promising candidate for rGO–Ag HNPT composites. In addition to the binding property, the equimolar concentration ratio of the rGO–Ag HNPT dosage has produced a remarkable antitumor efficacy. As a result, it might be applied to the development of the therapeutic drugs for SiHa cervical cells. (Figure 10).

## 4. Conclusion

The Ag hexagonal NPT-decorated rGO was created utilizing a straightforward one-step *in situ* synthesis process that did not require the use of any external templates. UV-visible spectrophotometer, HRTEM, EDAX, Raman, and XRD analyses were used to characterize the resulting nanocomposite. The mechanism for the production of Ag HNPTs on the rGO surface was proposed. Furthermore, the cytotoxic effects of rGO–Ag HNPT composites at various concentrations on SiHa cervical cancer cells were carefully examined. The determined half-maximum inhibitory concentration (IC_50_) for the manufactured nanocomposite against SiHa cells was 0.5 *μ*m. As a result, the rGO–Ag HNPT composites have an excellent cytotoxic impact and have the potential to be employed as an anticancer medication.

## Data Availability

All the data relevant to the manuscript are available on request from the first and corresponding authors.
